# A novel inflammatory score predicts hematoma expansion and 90‑day functional outcomes after spontaneous intracerebral hemorrhage

**DOI:** 10.1016/j.clinsp.2026.101004

**Published:** 2026-06-01

**Authors:** Xianxian Li, Yuchao Jia, Jiahe Ye, Xin Liu, Yuanbing Lu, Haoyi Chen, Dan Zhang, Xiaodong Ye, Ruizhi Xiao, Suiqiang Zhu, Shanshan Huang

**Affiliations:** Department of Neurology, Tongji Hospital, Tongji Medical College, Huazhong University of Science and Technology, China

**Keywords:** Intracerebral hemorrhage, Hematoma expansion, Inflammatory score, Systemic Immune-Inflammation Index, Risk stratification

## Abstract

**Background:**

Intracerebral Hemorrhage (ICH) entails high morbidity and rapid clinical deterioration; early risk stratification is critical. Although inflammatory biomarkers correlate with Hematoma Expansion (HE) and outcomes, their incremental value over existing models is uncertain.

**Methods:**

The authors retrospectively included consecutive acute ICH admissions to Tongji Hospital (2012‒2024). An inflammatory score (0‒9) was derived from the first post-admission blood draw (on admission or next morning), all within 24 h of admission, using neutrophil-to-lymphocyte ratio, platelet-to-lymphocyte ratio, monocyte-to-lymphocyte ratio, systemic immune-inflammation index (neutrophil × platelet/lymphocyte), lactate dehydrogenase, and high-sensitivity C-reactive protein. HE was defined as > 6 mL or > 33% on 24‒48 h follow-up Computed Tomography (CT). 90-day outcomes were poor function (modified Rankin Scale [mRS] score 4‒6) and mortality. Multivariable models used multiple imputation.

**Results:**

HE analyses included 1047 patients with baseline and 24‒48 h follow-up CT; 298 (28.5%) had HE. Outcome analyses included 953 patients (94-lacked 90-day follow-up); 399 (41.9%) had mRS 4‒6 and 120 (12.6%) died. The score was independently associated with HE (adjusted Odds Ratio 1.18), poor function (1.27), and mortality (1.30). Adding the score increased the area under the receiver operating characteristic curve (AUC) for HE (0.681 to 0.709; ΔAUC = 0.029; p = 0.007), poor function (0.826 to 0.842; ΔAUC = 0.016; p = 0.014), and mortality (0.815 to 0.838; ΔAUC = 0.022; p = 0.036). Sensitivity analyses, including a ≤ 6 h landmark analysis, inverse probability weighting (IPW) for follow-up availability, and complete-case analyses, yielded similar effects.

**Conclusion:**

An admission laboratory-based inflammatory score provides independent, incremental prognostic value for HE and 90-day outcomes after ICH.

**Registration-URL:**

http://www.chictr.org.cn. Unique identifier: ChiCTR-ROC-2,000039365.

## Highlights

• Six admission lab markers form a score predicting HE and 90-day outcomes.
• Score improves discrimination, calibration, and reclassification over base models.
• Prognostic value remains robust in landmark and IPW analyses.
• Decision-curve analysis shows net benefit across clinically relevant thresholds.

## Introduction

Intracerebral Hemorrhage (ICH) is a major subtype of stroke and is associated with high mortality and long-term disability.^1^ Early Hematoma Expansion (HE) occurs in approximately one-third of patients and is a key driver of clinical deterioration and poor outcomes.^2^ Accordingly, early risk stratification is essential for optimizing acute management.

Multiple predictive models have been developed for HE,^3^^,^^4^ incorporating clinical factors (e.g., baseline ICH volume, onset-to-imaging time, anticoagulant/antiplatelet use), imaging findings (e.g., Non-Contrast Computed Tomography [NCCT] hypodensity or Computed Tomography Angiography [CTA] spot sign), and basic laboratory parameters.^5^^,^^6^ However, most models do not incorporate systemic inflammatory biomarkers.

Prognostic models for ICH outcomes, such as the ICH score.^7^ max-ICH score.^8^ ICH Functional Outcome Score,^9^ ICH Grading Scale,^10^ ICH Outcomes Project score,^11^ and ADVISING score.^12^ primarily include demographic and clinical predictors such as age, hematoma volume, location, and level of consciousness (e.g., Glasgow Coma Scale [GCS] or National Institutes of Health Stroke Scale [NIHSS]). Although some models have explored inflammatory markers as additional predictors, their incremental contribution to model performance remains underexplored. Moreover, most prior studies evaluate single inflammatory markers in isolation, whereas a composite score may better capture the multidimensional systemic inflammatory response and reduce reliance on any one unstable biomarker.

Recent evidence suggests that integrating multiple routinely available inflammatory indices into a composite score, including the Neutrophil-to-Lymphocyte Ratio (NLR), Platelet-to-Lymphocyte Ratio (PLR), Monocyte-to-Lymphocyte Ratio (MLR), Systemic Immune-Inflammation Index (SII; neutrophil × platelet/lymphocyte), Lactate Dehydrogenase (LDH), and high-sensitivity C-Reactive Protein (hsCRP), is associated with HE and adverse outcomes after ICH.^13^

Accordingly, the authors evaluated whether an admission laboratory-based composite inflammatory score improves prediction when added to established models for HE and 90-day outcomes after ICH.

## Methods

### Patients

This study included consecutive ICH patients treated at Tongji Hospital, Tongji Medical College, Huazhong University of Science and Technology, China, from January 2012 to October 2024. Inclusion criteria were as follows: 1) Age ≥ 18-years; 2) Acute onset of neurologic or systemic symptoms (e.g., headache, vomiting); 3) CT revealing ICH; and 4) Initial CT was conducted within 6-hours of symptom onset, with a follow-up CT performed 24- to 48-hours later. Exclusion criteria were: 1) Primary ventricular hemorrhage; 2) Hemorrhage due to brain tumors, hemorrhagic cerebral infarction, traumatic brain injury, or multiple hemorrhages; 3) No repeat head CT within 24- to 48-hours of the initial CT (including discharge within 24 h, surgical intervention before follow-up CT, unable to be weaned from mechanical ventilation, and other subjective reasons due to patient and relatives; see Supplementary Fig. S1); and 4) Missing all admission laboratory data required to derive the inflammatory score.

This retrospective cohort study was designed and reported in accordance with the Strengthening the Reporting of Observational Studies in Epidemiology (STROBE) guidelines. Ethical approval for this study was obtained from the Institutional Ethics Committee of Huazhong University of Science and Technology (approval n° [2020] Lun Shen Zi [S145]). All procedures involving human participants complied with the Declaration of Helsinki and relevant institutional regulations. Written informed consent was obtained from all participants before inclusion. The study protocol was registered with the Chinese Clinical Trial Registry on 24 October 2020 (ChiCTR-ROC2000039365).

### Inflammatory score construction

The authors prespecified a six-biomarker inflammatory score comprising NLR, PLR, MLR, SII, LDH, and hsCRP, yielding 0‒9 points. Ratio-based components were dichotomized using site-specific thresholds computed from each laboratory’s reference intervals (Upper/Lower Normal Limits [UNL/LNL]) according to prespecified formulas (Supplementary Table S1). The numeric cutoffs reported for the studied center are provided for transparency only and should not be transported verbatim; external implementation should recompute thresholds by substituting the local upper and lower normal limits into the same formulas (Supplementary Table S1). In the studied center, these were NLR ≥ 5.25, PLR ≥ 184, MLR ≥ 0.50, SII ≥ 1050, LDH ≥ 225 U/L, and hsCRP ≥ 10 mg/L. Scoring rules were fixed: among the four ratio-based indicators (NLR, PLR, MLR, SII), the first positive indicator contributed 2-points, and each additional positive indicator contributed 1-point (maximum 5-points from ratio-based components). LDH and hsCRP each contributed 2-points when above threshold.

### Clinical variables

Data collected included medical and medication history, laboratory tests, clinical examinations, blood pressure, NIHSS score at admission, and treatment modalities. Laboratory values for score derivation were from the first post-admission blood draw (on admission or next morning), all within 24 h of admission. All CT images were reviewed by two neurologists, with interpretations based on the radiologist's report.

### Outcome

HE was defined as an absolute increase of hematoma volume > 6 mL or a relative increase > 33% on follow-up CT within 24 to 48 hours compared to the first CT. Outcome measures included death (mRS = 6) and functional dependence (mRS > 3) within 3-months, with mRS scores obtained via phone or text from patients or close care relatives during the follow-up.

### Statistical analysis

Categorical variables were presented as n (%) and compared by χ^2^ tests; continuous variables were summarized as mean ± SD or median (IQR) and compared with *t*-tests or Mann-Whitney *U*-tests, as appropriate. The authors modeled HE and 90-day outcomes (mRS ≥4 and mortality) using multivariable logistic regression, with two prespecified specifications per endpoint: a clinical base model and the same model plus the inflammatory score. For HE, the 5-predictor base model included prior anticoagulant use, antiplatelet use, onset-to-baseline CT time, baseline hematoma volume, and hypodensities on the baseline CT.^5^ the augmented model added the inflammatory score. For mRS and mortality, the base “max-ICH components” model comprised age, NIHSS on admission, baseline volume, lobar location, Intraventricular Hemorrhage (IVH), and prior anticoagulation,^8^ with an augmented model that additionally included the inflammatory score.

Missing components of the inflammatory score (white blood cell, neutrophil, lymphocyte, monocyte, and platelet counts; hsCRP; LDH) were multiply imputed using multivariate imputation by chained equations (MICE; m = 20) restricted to analysis rows; only imputed values were constrained to plausible bounds derived from the observed distribution, and observed values were never truncated. All non-laboratory covariates were required to be complete on analysis rows, and complete-case sensitivity analyses were performed.

To address bias, HE analyses corrected both selection and timing. The authors evaluated baseline balance with standardized mean differences and then applied stabilized Inverse Probability Weighting (IPW) for receipt of a 24‒48 h follow-up non-contrast CT (CT-IPW) in the combined inception cohort (with and without follow-up CT), using only pre-decision variables (age, sex, hypertension, diabetes, hyperlipidemia, ischemic heart disease, antiplatelet/anticoagulant use, prior ischemic stroke or ICH, smoking, alcohol use, systolic/diastolic blood pressure, NIHSS, baseline volume, lobar location, IVH); weights were stabilized and truncated at the 99th percentile. The authors mitigated immortal-time bias using a binary indicator of phlebotomy within ≤ 6-hours of symptom onset via a ≤ 6-hour landmark restriction and stabilized IPW for Early Phlebotomy (E-IPW) based on the same pre-decision covariates as CT-IPW plus onset-to-CT time and hypodensities; when both mechanisms applied, combined weights (CT-IPW × E-IPW) were used. The ≤ 6 h landmark analysis was prespecified as a conservative sensitivity analysis to enforce temporal ordering, aligning with the study’s inception window requiring baseline CT within 6-hours of symptom onset. For mRS and mortality, only timing bias was addressed using the same ≤ 6-hour landmark restriction and E-IPW; no CT-IPW was applied.

Model performance was summarized by the Area Under the receiver operating characteristic Curve (AUC) with 95% confidence intervals, DeLong-tested ΔAUC, and the Brier score; operating characteristics were reported at prespecified risk thresholds (0.10, 0.20, 0.30). Incremental clinical value was quantified by continuous Net Reclassification Improvement (NRI) and Integrated Discrimination Improvement (IDI). Decision Curve Analysis (DCA) was used to compare the net benefit of the base versus score-augmented models across threshold probabilities of 0.05–0.35, encompassing the prespecified operating points. Primary analyses used multiple imputation (m = 20); sensitivity analyses included complete-case analyses. All statistical analyses were performed in IBM SPSS 26.0 and R 4.5.1.

## Results

**Fig. 1:**
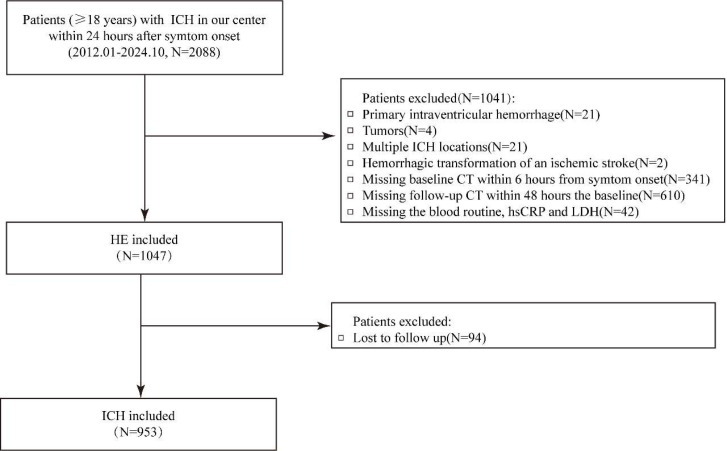
Selection flowchart.

Among 1047 patients with both baseline and follow-up CT, 298 (28.5%) had HE. After excluding 94 patients without 90-day follow-up, 953 remained for outcome analyses; 399 (41.9%) had poor functional outcome (mRS 4–6) and 120 (12.6%) died. Cohort selection is shown in Fig. 1. Baseline summaries used observed values only (no imputation). Compared with non-HE patients, those with HE had larger baseline hematoma volumes, shorter onset-to-CT times, more antiplatelet/anticoagulant use, more NCCT hypodensities and IVH, higher NIHSS scores, and higher inflammatory scores (Table 1). For outcomes, patients with mRS 4‒6 or death were older and had higher NIHSS scores and baseline volumes, more IVH, and higher inflammatory scores (Table 2). Baseline characteristics of included versus excluded patients are reported in Supplementary Table S2. Missingness in the imputed biomarkers was low (≤1.9%). In the HE cohort, missingness was 0.57% for white blood cell, 0.57% for neutrophils, 0.67% for lymphocytes, 0.57% for monocytes, 0.38% for platelets, 1.91% for hsCRP, and 1.43% for LDH; corresponding proportions in the 3-month outcome cohort were 0.52%, 0.52%, 0.63%, 0.52%, 0.31%, 1.68%, and 1.15%, respectively (Supplementary Table S3).

**Table 1:** Comparison of baseline characteristics in patients with and without hematoma expansion.

	**Total** **(n = 1047)**	**Non-hematoma expansion** **(n = 749)**	**Hematoma expansion (n = 298)**	**p-value**
**Male sex, n (%)**	710 (67.8%)	496 (66.2%)	214 (71.8%)	0.081
**Age, mean ± SD, y**	56.21 ± 12.51	56.3 ± 12.38	56.97 ± 12.87	0.696
**Hyertension, n (%)**	761 (72.7%)	551 (73.6%)	210 (70.5%)	0.311
**Diabetes mellitus, n (%)**	113 (10.8%)	72 (9.6%)	41 (13.8%)	0.051
**Ischemic heart disease, n (%)**	66 (6.3%)	44 (5.9%)	22 (7.4%)	0.365
**Hyperlipidemia, n (%)**	65 (6.2%)	42 (5.6%)	23 (7.7%)	0.202
**Antiplatelets use, n (%)**	45 (4.3%)	24 (3.2%)	21 (7.0%)	**0.006**
**Anticoagulants use, n (%)**	39 (3.7%)	20 (2.7%)	19 (6.4%)	**0.004**
**Prior ischemic/ICH stroke, n (%)**	153 (14.6%)	108 (14.4%)	45 (15.1%)	0.778
**Smoking, n (%)**	357 (34.1%)	256 (34.2%)	101 (33.9%)	0.930
**Alcohol use, n (%)**	328 (31.3%)	234 (31.2%)	94 (31.5%)	0.924
**Systolic blood pressure, mean ± SD, mmHg**	155.62 ± 23.62	155.77 ± 24.19	155.24 ± 22.16	0.743
**Diastolic blood pressure, mean ± SD, mmHg**	91.27 ± 16.12	91.01 ± 16.41	91.9 ± 15.36	0.421
**White blood cell count, median (IQR), 10^10/L**	9.65 (7.35‒12.47)	9.47 (7.34‒12.19)	10.37 (7.39‒13.03)	0.050
**Neutrophils count, median (IQR), 10^10/L**	7.95 (5.59‒10.85)	7.69 (5.44‒10.31)	8.54 (6.02‒11.38)	**0.008**
**Lymphocytes count, median (IQR), 10^10/L**	1.11 (0.79‒1.53)	1.16 (0.83‒1.57)	1.03 (0.69‒1.39)	**<0.001**
**Monocytes count, median (IQR), 10^10/L**	0.51 (0.37‒0.69)	0.5 (0.37‒0.68)	0.53 (0.39‒0.73)	0.137
**Hb, median (IQR), g/L**	141 (129‒152)	141(128‒150)	142 (129‒153)	0.096
**PLT, median (IQR), 10^9/L**	204 (165‒242)	204 (169‒246)	203 (157‒238)	0.096
**hsCRP, median (IQR), mg/L**	4.9 (1.6‒13.5)	4.3 (1.5‒10.7)	7.35 (2‒22.58)	**<0.001**
**LDH, median (IQR), U/L**	208 (180‒244)	204 (178‒239)	213.5 (186.75‒250)	**0.006**
**ALT, median (IQR), U/L**	17 (12‒24)	16 (12‒24)	17 (13‒25)	**0.045**
**AST, median (IQR), U/L**	21 (17‒26)	20 (17‒26)	21 (17‒26)	0.318
**Creatinine, median (IQR), μmoL/L**	304.25 (234.63‒382.85)	304 (238.7‒382.8)	304.5 (226.2‒383)	0.740
**Uric acid, median (IQR), mmoL/L**	72 (58‒89)	72 (58‒90)	73 (58‒88)	0.866
**International normalized ratio, median (IQR)**	1.01 (0.96‒1.07)	1.01 (0.96‒1.07)	1.01 (0.96‒1.07)	0.618
**Glucose level, median (IQR), mmoL/L**	6.57 (5.63‒8.03)	6.38 (5.56‒7.61)	7.01 (5.87‒8.98)	**<0.001**
**NIHSS on admission, median (IQR), score**	11 (6‒17)	10 (5.5‒16)	14 (9‒22)	**<0.001**
**Time from symptom onset to CT, median (IQR), h**	3 (2‒5.5)	3.5 (2‒6)	2.5 (1.5‒4.5)	**<0.001**
**ICH location, n (%)**				0.180
Lobar	272 (26%)	186 (24.8%)	86 (28.9%)	
Subcortical	775 (74%)	563 (75.2%)	212 (71.1%)	
**Baseline ICH volume, median (IQR), mL**	16.47 (7.31‒33.93)	15.54 (6.74‒30.82)	21.59 (10.47‒41.79)	**<0.001**
**NCCT hypodensities, n (%)**	171 (16.3%)	84 (11.2%)	87 (29.2%)	**<0.001**
**Presence of IVH, n (%)**	332 (31.7%)	222 (29.6%)	110 (36.9%)	**0.022**
**Inflammatory score, median (IQR), score**	5 (2‒6)	4 (3‒6)	5 (4‒7)	**<0.001**

Baseline characteristics are summarized from observed data only; imputed values were not used. Values are mean ± SD or median (IQR) as appropriate; categorical variables are n (%).

ALT, Alanine Aminotransferase; AST, Aspartate Aminotransferase; CT, Computed Tomography; Hb, Hemoglobin; hsCRP, high-sensitivity C-Reactive Protein; ICH, Intracerebral Hemorrhage; IQR, Interquartile Range; IVH, Intraventricular Hemorrhage; LDH, Lactate Dehydrogenase; NCCT, Non-Contrast Computed Tomography; NIHSS, National Institute of Health stroke scale; p-value, Probability value; SD, Standard Deviation.

**Table 2:** Univariate analysis comparing patients with good and poor outcomes.

	**Total** **(n = 953)**	**3‒month mRS 0‒3 (n = 554)**	**3‒month mRS 4‒6** **(n = 399)**	**p‒value**	**3‒month survial** **(n = 833)**	**3‒month morality (n = 120)**	**p-value**
**Male sex, n (%)**	646 (67.8%)	379 (68.4%)	267 (66.9%)	0.626	563 (67.6%)	83 (69.2%)	0.729
**Age, mean ± SD, y**	56.14 ± 12.63	54.48 ± 11.75	58.44 ± 13.44	**<0.001**	56.41 ± 12.36	61.20 ± 13.39	**<0.001**
**Hypertension, n (%)**	693 (72.7%)	392 (70.8%)	301 (75.4%)	0.11	606 (72.7%)	87 (72.5%)	0.954
**Diabetes mellitus, n (%)**	109 (11.4%)	56 (10.1%)	53 (13.3%)	0.129	86 (10.3%)	23 (19.2%)	**0.004**
**Ischemic heart disease, n (%)**	62 (6.5%)	28 (5.1%)	34 (8.5%)	**0.032**	49 (5.9%)	13 (10.8%)	**0.040**
**Hyperlipidemia, n (%)**	60 (6.3%)	35 (6.3%)	25 (6.3%)	0.974	48 (5.8%)	12 (10%)	0.074
**Antiplatelets use, n (%)**	45 (4.7%)	23 (4.2%)	22 (5.5%)	0.328	37 (4.4%)	8(6.7%)	0.283
**Anticoagulants use, n (%)**	37 (3.9%)	20 (3.6%)	17 (4.3%)	0.608	32 (3.8%)	5 (4.2%)	0.863
**Prior ischemic/ICH stroke, n (%)**	141 (14.8%)	70 (12.6%)	71 (17.8%)	**0.027**	116 (13.9%)	25 (20.8%)	**0.046**
**Smoking, n (%)**	319 (33.5%)	191 (34.5%)	128 (32.1%)	0.439	287 (34.5%)	32 (26.7%)	0.091
**Alcohol use, n (%)**	293 (30.7%)	184 (33.2%)	290 (27.3%)	0.052	265 (31.8%)	28 (23.3%)	0.060
**Systolic blood pressure, mean ± SD, mmHg**	155.69 ± 23.45	154.25 ± 23.4	157.7 ± 23.41	**0.025**	155.5 ± 23.29	157.05 ± 24.6	0.500
**Diastolic blood pressure, mean ± SD, mmHg**	91.31 ± 16.25	91.67 ± 16.31	90.8 ± 16.18	0.412	91.61 ± 16.17	89.22 ± 16.71	0.133
**White blood cell count, median (IQR), 10^9/L**	9.65 (7.34‒12.47)	8.92 (7.07‒11.44)	10.72 (8.25‒13.85)	**<0.001**	9.3 (7.29‒11.9)	12.75 (9.64‒15.65)	**<0.001**
**Neutrophils count, median (IQR), 10^9/L**	7.97 (5.58‒10.88)	7.04 (5.01‒9.56)	9.04 (6.47‒11.93)	**<0.001**	7.51 (5.42‒10.07)	11.41 (8.17‒14)	**<0.001**
**Lymphocytes count, median (IQR), 10^9/L**	1.11 (0.8‒1.53)	1.23 (0.9‒1.68)	0.98 (0.65‒1.36)	**<0.001**	1.16 (0.83‒1.57)	0.81 (0.57‒1.11)	**<0.001**
**Monocytes count, median (IQR), 10^9/L**	0.51 (0.37‒0.69)	0.5 (0.36‒0.66)	0.53 (0.4‒0.74)	**0.002**	0.5 (0.37‒0.68)	0.56 (0.4‒0.75)	**0.021**
**PLT, median (IQR), 10^9/L**	206 (166‒244)	208.5 (170‒250)	200 (160.75‒234)	**0.011**	206 (167.75‒245)	199 (155‒232.25)	0.102
**hsCRP, median (IQR), mg/L**	5 (1.65‒13.8)	3.9 (1.25‒8.9)	7.75 (2.5‒29.08)	**<0.001**	4.5 (1.5‒12.53)	9.8 (3.6‒35.2)	**<0.001**
**LDH, median (IQR), U/L**	208.5 (180‒245)	201 (174‒228)	225 (195‒264)	**<0.001**	206 (178‒239.25)	230 (200.5‒268)	**<0.001**
**Hb, median (IQR), g/L**	141 (128‒152)	141 (129‒152)	140 (126‒151.5)	**0.181**	141 (129‒152)	13 9 (122‒150)	0.094
**ALT, median (IQR), U/L**	16 (12‒24)	17 (12‒24)	16 (12‒24)	0.599	16 (12‒24)	17 (12‒24.75)	0.722
**AST, median (IQR), U/L**	21 (17‒26)	20 (16‒26)	22 (17‒27)	**0.002**	20 (17‒26)	22 (17‒29)	0.080
**Creatinine, median (IQR), µmoL/L**	304 (237‒385)	307 (245.9‒390.35)	294.3 (226‒382)	0.275	300.8 (237‒382)	321.5 (230.18‒413.4)	0.092
**Uric acid, median (IQR), mmoL/L**	73 (58‒90)	72 (59‒88)	74.5 (58‒91)	0.093	72 (58‒88)	80.5 (62‒102.25)	**0.001**
**International normalized ratio, median IQR)**	1.01 (0.96‒1.07)	1.01(0.95‒1.06)	1.02 (0.97‒1.08)	**0.001**	1.01 (0.95‒1.07)	1.03 (0.99‒1.09)	**<0.001**
**Glucose level, median (IQR), mmoL/L**	6.58 (5.66‒8.09)	6.23 (5.43‒7.25)	7.26 (6.07‒9.32)	**<0.001**	6.42 (5.56‒7.63)	8.67 (6.87‒11.57)	**<0.001**
**NIHSS on admission, median (IQR), score**	11 (6‒17)	8 (4‒12)	16 (11‒27)	**<0.001**	10 (6‒15)	22 (12.25‒32)	**<0.001**
**ICH location, n (%)**				0.545			**0.025**
Lobar	253 (26.5%)	143 (25.8%)	110 (27.6%)		211 (25.3%)	42 (35%)	
Subcortical	700 (73.5%)	411 (74.2%)	289 (72.4%)		622 (74.7%)	78 (65%)	
**Baseline ICH volume, median (IQR), mL**	16.38 (7.2‒34.06)	12.41 (5.54‒26.79)	25.88 (10.94‒45.13)	**<0.001**	15.41 (6.87‒30.58)	33.72 (15.29‒58.09)	**<0.001**
**Presence of IVH, n (%)**	291 (30.5%)	122 (22%)	169 (42.4%)	**<0.001**	236 (28.3%)	55 (45.8%)	**<0.001**
**Treatment, n (%)**				**<0.001**			**0.001**
Medical treatment	677 (71%)	443 (80%)	234 (58.6%)		607 (72.9%)	70 (58.3%)	
Surgical management	276 (29%)	111 (20%)	165 (41.4%)		226 (27.1%)	50 (41.7%)	
**Inflammatory score, median (IQR), score**	5 (2‒6)	5 (2‒5)	5 (5‒7)	**<0.001**	4 (2‒6)	7 (5‒7)	**<0.001**

Baseline characteristics are summarized from observed data only; imputed values were not used. Values are mean±SD or median (IQR) as appropriate; categorical variables are n (%).

ALT, Alanine Aminotransferase; AST, Aspartate Aminotransferase; CT, Computed Tomography; Hb, Hemoglobin; hsCRP, High-Sensitivity C-Reactive Protein; ICH, Intracerebral Hemorrhage; IQR, Interquartile Range; IVH, Intraventricular Hemorrhage; LDH, Lactate Dehydrogenase; mRS, modified Rankin Scale; NCCT, Non-Contrast Computed Tomography; NIHSS, National Institute of Health Stroke Scale; p-value, Probability value; SD, Standard Deviation.

**Fig. 2:**
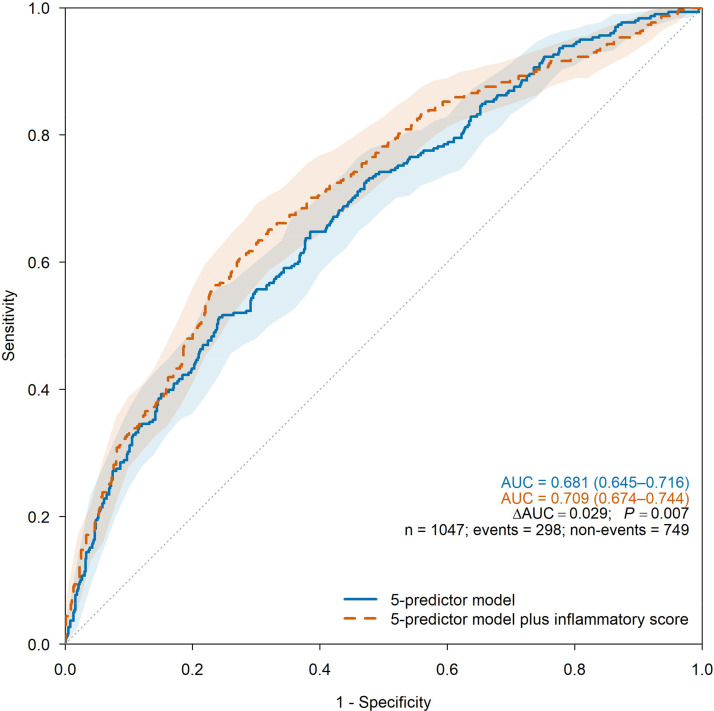
ROC curves predicting hematoma expansion (n = 1047).

In multivariable models after multiple imputation, the inflammatory score was independently associated with HE (Adjusted Odds Ratio [AOR = 1.18], 95% CI 1.11‒1.26; Table 3) and improved the 5-predictor HE model (AUC increased from 0.681 [0.645‒0.716] to 0.709 [0.674‒0.744]; ΔAUC = 0.029; DeLong *P* = 0.007; Brier decreased from 0.185 to 0.179; cfNRI = 0.382; IDI = 0.029; Table 4; Fig. 2). Complete-case results were similar (Supplementary Fig. S2; Tables S4‒S5). Under CT-IPW, standardized mean differences indicated adequate balance between patients with and without follow-up CT (Supplementary Table S6). Landmark (≤ 6 h) and IPW sensitivity analyses showed consistent, slightly attenuated gains: unweighted ΔAUC = 0.024 (DeLong *P* = 0.059), E-IPW ΔAUC = 0.024 (p = 0.070), CT-IPW ΔAUC = 0.025 (p = 0.053), and combined IPW ΔAUC = 0.024 (p = 0.061) (Supplementary Table S7). At the prespecified HE threshold of 0.30, the augmented model yielded sensitivity/specificity of 60.4%/72.6% (Supplementary Table S8), with comparable complete-case performance. Multicollinearity was minimal (maximum variance inflation factors ≤ 1.509), and results were consistent in sensitivity analyses excluding baseline hematoma volume (Supplementary Tables S9‒S10). DCA showed higher net benefit for the augmented HE model across clinically plausible thresholds (0.05–0.35) (Supplementary Fig. S3).

**Table 3:** Adjusted odds ratios for hematoma expansion after multiple imputation with and without the inflammatory score.

**Variable**	**HE (n = 1047)**	
	**Adjusted Odds ratio (95% CI)**	**p-value**
**5-predictor model**		
Antiplatelets use, n (%)	2.36 (1.21–4.62)	0.012
Anticoagulants use, n (%)	2.43 (1.30–4.56)	0.006
Time from symptom onset to CT, h	0.83 (0.77–0.90)	<0.001
Baseline ICH volume, mL	1.01 (1.00–1.02)	<0.001
NCCT hypodensities, n (%)	3.11 (2.19–4.42)	<0.001
**5-predictor model plus inflammatory score**		
Antiplatelets use, n (%)	2.72 (1.37–5.41)	0.004
Anticoagulants use, n (%)	2.67 (1.40–5.08)	0.003
Time from symptom onset to CT, h	0.83 (0.77–0.90)	<0.001
Baseline ICH volume, mL	1.00 (1.00–1.01)	0.124
NCCT hypodensities, n (%)	3.03 (2.12–4.33)	<0.001
Inflammatory score, score	1.18 (1.11–1.26)	<0.001

Seven laboratory variables were imputed using multiple imputation by chained equations (m = 20); estimates were pooled using Rubin’s rules. The inflammatory score components and definitions are provided in Supplementary Table S1.

CI, Confidence Interval; CT, Computed Tomography; HE, Hematoma Expansion; ICH, Intracerebral Hemorrhage; NCCT, Non-Contrast Computed Tomography.

Inflammatory score components: hsCRP, high-sensitivity C-Reactive Protein; LDH, Lactate Dehydrogenase; MLR, Monocyte-to-Lymphocyte Ratio; NLR, Neutrophil-to-Lymphocyte Ratio; PLR, Platelet-to-Lymphocyte Ratio; SII, Systemic Immune-Inflammation Index.

**Fig. 3:**
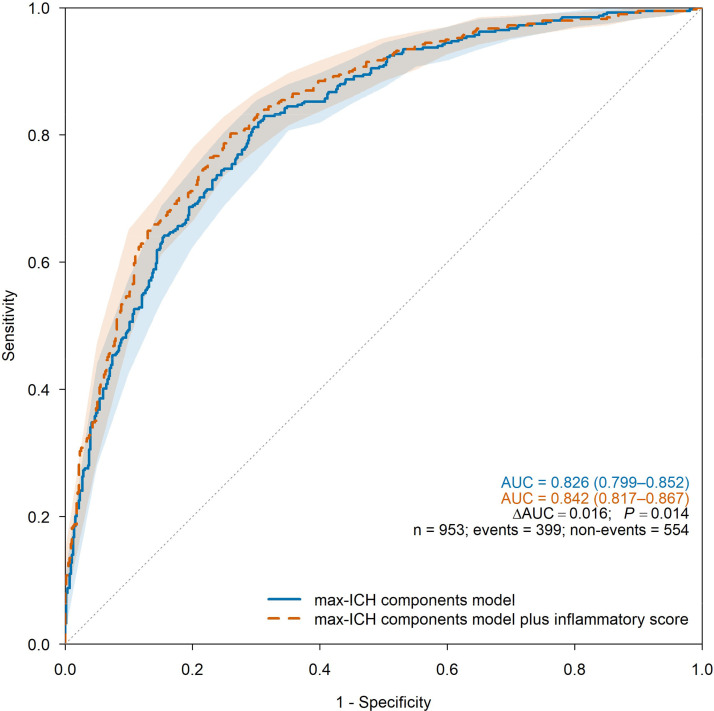
ROC curves for 3-month functional outcome (n = 953).

**Fig. 4:**
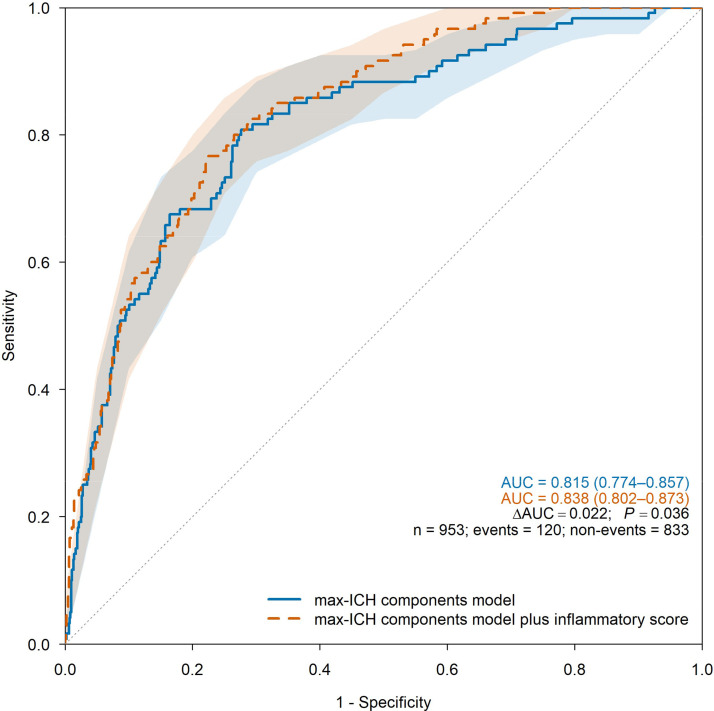
ROC curves predicting 3-month mortality (n = 953).

For 90-day outcomes, the inflammatory score remained independently associated with poor function (AOR = 1.27, 95% CI 1.18‒1.36) and mortality (AOR = 1.30, 95% CI 1.17‒1.44) in the max-ICH components model (Table 5) and improved discrimination: mRS 4‒6 AUC increased from 0.826 [0.799‒0.852] to 0.842 [0.817‒0.867] (ΔAUC = 0.016; p = 0.014; Brier decreased from 0.169 to 0.159; cfNRI = 0.450; IDI = 0.039) and mortality AUC increased from 0.815 [0.774‒0.857] to 0.838 [0.802‒0.873] (ΔAUC = 0.022; p = 0.036; Brier decreased from 0.091 to 0.087; cfNRI = 0.546; IDI = 0.030; Table 4; Figs. 3,4). DCA similarly favored the augmented models for mRS 4–6 and mortality across thresholds of 0.05–0.35 (Supplementary Fig. S3). Complete-case analyses were similar (Supplementary Tables S5 and S11; Figs. S4‒S5). Landmark analyses were concordant, with a significant ΔAUC for mRS 4‒6 and a directionally similar, non-significant ΔAUC for mortality (Supplementary Table S12). At prespecified thresholds, the augmented models achieved sensitivity/specificity of 85.7%/65.0% for mRS 4‒6 at 0.30 and 82.5%/70.0% for mortality at 0.10 (Supplementary Table S8), again with comparable complete-case performance.

**Table 4:** Model performance after multiple imputation for hematoma expansion and 3-month modified Rankin Scale outcomes.

**Model**	**AUC (95% CI)**	**ΔAUC**	**DeLong *P***	**Wald *P***	**LRT *P***	**Brier**	**cfNRI (95% CI; p)**	**IDI (95% CI; p)**
**HE (n = 1047)**								
5-predictor model	0.681 (0.645‒0.716)					0.185		
5-predictor model plus Inflammatory Score	0.709 (0.674‒0.744)	0.029	0.007	<0.001	NA	0.179	0.382 (0.290‒0.474; <0.001)	0.029 (0.019‒0.039; <0.001)
**3-month mRS 4‒6 (n = 953)**								
max-ICH components model	0.826 (0.799‒0.852)					0.169		
max-ICH components model plus inflammatory score	0.842 (0.817‒0.867)	0.016	0.014	<0.001	NA	0.159	0.450 (0.362‒0.539; <0.001)	0.039 (0.026‒0.051; <0.001)
**3-month mRS 6 (n = 953)**								
max-ICH components model	0.815 (0.774‒0.857)					0.091		
max-ICH components model plus inflammatory score	0.838 (0.802‒0.873)	0.022	0.036	<0.001	NA	0.087	0.546 (0.417‒0.675; <0.001)	0.030 (0.015‒0.044; <0.001)

AUC, Area Under the receiver operating Characteristic Curve; Brier, Brier score (mean squared error of probabilistic predictions; lower values indicate better accuracy); CI, Confidence Interval; cfNRI, category-free net reclassification improvement (i.e., continuous NRI that does not require prespecified risk categories); DeLong *P*, two-sided p-value from DeLong’s test comparing paired AUCs; ΔAUC, difference in AUC between the augmented model (with the inflammatory score) and the base model; IDI, Integrated Discrimination Improvement; LRT *P*, p-value from the likelihood-ratio test (not applicable for multiply imputed models); NA, Not Applicable.

**Table 5:** Adjusted odds ratios for 3-month modified Rankin Scale outcomes after multiple imputation for models with and without the inflammatory score.

**Variable**	**3-month mRS 4‒6 (n = 953)**		**3-month mRS 6 (n = 953)**	
	**Adjusted Odds ratio (95% CI)**	**p-value**	**Adjusted Odds ratio (95% CI)**	**p-value**
**max-ICH components model**				
Age, year	1.04 (1.03–1.05)	<0.001	1.05 (1.03–1.06)	<0.001
Anticoagulants use, n (%)	0.78 (0.35–1.71)	0.527	0.79 (0.28–2.24)	0.660
NIHSS on admission, score	1.13 (1.11–1.16)	<0.001	1.09 (1.07–1.12)	<0.001
ICH location (lobar), n (%)	1.45 (0.97–2.17)	0.074	0.99 (0.59–1.68)	0.979
Baseline ICH volume, mL	1.02 (1.01–1.03)	<0.001	1.01 (1.00–1.02)	0.008
Presence of IVH, n (%)	1.85 (1.32–2.59)	<0.001	1.27 (0.81–1.99)	0.302
**max-ICH components model plus inflammatory score**				
Age, year	1.04 (1.03–1.06)	<0.001	1.05 (1.03–1.06)	<0.001
Anticoagulants use, n (%)	1.03 (0.46–2.30)	0.947	0.98 (0.33–2.92)	0.975
NIHSS on admission, score	1.12 (1.09–1.14)	<0.001	1.08 (1.06–1.10)	<0.001
ICH location (lobar), n (%)	1.57 (1.03–2.38)	0.035	1.10 (0.64–1.88)	0.728
Baseline ICH volume, mL	1.01 (1.00–1.02)	0.008	1.01 (1.00–1.02)	0.053
Presence of IVH, n (%)	1.72 (1.22–2.44)	0.002	1.19 (0.75–1.88)	0.454
Inflammatory score, score	1.27 (1.18–1.36)	<0.001	1.30 (1.17–1.44)	<0.001

Multiple imputation of seven laboratory variables was performed with 20 imputations; estimates were pooled using Rubin’s rules. The inflammatory score components and definitions are provided in Supplementary Table S1.

CI, Confidence Interval; ICH, Intracerebral Hemorrhage; IVH, Intraventricular Hemorrhage; mRS, modified Rankin Scale; NIHSS, National Institutes of Health Stroke Scale.

Inflammatory score components: hsCRP, high-sensitivity C-Reactive Protein; LDH, Lactate Dehydrogenase; MLR, Monocyte-to-Lymphocyte Ratio; NLR, Neutrophil-to-Lymphocyte Ratio; PLR, Platelet-to-Lymphocyte Ratio; SII, Systemic Immune-Inflammation Index.

## Discussion

An inflammatory score was independently associated with Hematoma Expansion (HE) and 90-day outcomes after Intracerebral Hemorrhage (ICH). When added to the 5-predictor HE-model and the max-ICH components model, the inflammatory score provided incremental prognostic value. Discrimination improved modestly but significantly (ΔAUC = 0.029 for HE; 0.022 for mortality; 0.016 for mRS 4‒6). It also lowered Brier scores and improved reclassification. At prespecified thresholds (HE = 0.30; mRS 4‒6: 0.30; mortality 0.10), sensitivity/specificity were 60.4%/72.6%, 85.7%/65.0%, and 82.5%/70.0%, respectively. Although the ΔAUC gains were modest, consistent improvements in calibration/reclassification and net benefit across clinically relevant thresholds suggest potential utility for risk stratification and guiding follow-up imaging in ICH.

After ICH, secondary injury is driven by blood product-triggered inflammatory and oxidative cascades. These cascades activate resident glia and recruit peripheral immune cells, potentially disrupting the blood-brain barrier and worsening perihematomal edema.^14^, ^15^, ^16^, ^17^ This provides biological plausibility for the observed associations between systemic inflammatory activity, hematoma expansion, and 90-day outcomes. The authors emphasize that this study is observational and addresses prognosis, not treatment effects.

Integrating inflammatory biomarkers into prediction models has been proposed but remains uncommon. Established ICH models (e.g., ICH score and max-ICH) primarily rely on age, hematoma volume/location, and level of consciousness, with occasional inclusion of routine admission laboratories.^7^, ^8^, ^9^, ^10^, ^11^, ^12^ HE models emphasize clinical risk factors^6^ and NCCT/CTA imaging markers.^5^^,^^18^ Biomarkers are used less often and typically reflect coagulation/hemostasis or inflammation/vascular injury. Examples include MMP-9,^19^ interleukin-6 (IL-6),^20^ hsCRP,^21^ and LDH.^22^ Evidence for single biomarkers is fragmented and rarely externally validated, limiting their uptake in multivariable tools. The authors therefore evaluated whether a composite inflammatory score adds prognostic value beyond established HE and outcome models.

The inflammatory score is a promising choice for inclusion in prediction models. It includes NLR, PLR, MLR, SII, LDH, and hsCRP, all easily accessible in clinical practice.^13^ Calculation uses routine admission laboratories with a prespecified mapping of center-specific reference limits (UNL/LNL) to cutoffs and points. Full thresholds and derivation formulas are provided in Supplementary Table S1. Because absolute blood-cell counts vary with hydration, specimen handling, and population factors, the ratio-based components (NLR/PLR/MLR) help mitigate such variability and may yield more stable prognostic signals.^23^

In multiply imputed analyses (m = 20), adding the inflammatory score increased discrimination (ΔAUC = 0.029 for HE, 0.016 for mRS 4‒6, and 0.022 for mortality) and lowered Brier scores (HE = 0.185 to 0.179; mRS 0.169 to 0.159; mortality 0.091 to 0.087). Reclassification also improved (cfNRI/IDI: HE = 0.382/0.029; mRS 4‒6 0.450/0.039; mortality 0.546/0.030; all p < 0.001), indicating added information beyond clinical and imaging covariates. Complete-case estimates closely matched these results. Limiting laboratories to ≤ 6 h and before outcome ascertainment reduced magnitudes but not direction. CT-IPW (for follow-up imaging) and E-IPW (for early sampling) yielded similar patterns. Overall, the score contributes prognostic information largely orthogonal to existing predictors. Its practical value should depend on calibration and net benefit at clinically chosen thresholds and should be confirmed by external validation.

Several limitations warrant consideration. First, inflammatory markers were measured only once within 24 h of admission, precluding assessment of temporal dynamics. Second, outcomes were assessed at 3-months, and longer-term trajectories were unavailable. Third, HE ascertainment required a 24–48 h follow-up CT; thus, patients with early death or emergent surgery were less likely to undergo repeat imaging and may be underrepresented, even after CT-IPW. To mitigate potential immortal-time bias due to delayed phlebotomy, the authors applied a ≤ 6 h landmark restriction and E-IPW; residual bias may remain. Time-dependent modeling was not feasible because HE was determined over an interval rather than at an exact time point, and laboratory sampling times were coarsened. For external implementation, cutoffs should be recalibrated using local UNL/LNL reference limits (Supplementary Table S1). The single-center design, based on a large tertiary referral hospital in China, may limit generalizability to other populations and healthcare settings. Although inverse probability weighting and landmark analyses addressed selection and timing biases, residual confounding from unmeasured factors (e.g., severity of comorbid conditions) cannot be excluded. Missing laboratory values were imputed under a missing-at-random assumption; although low missingness and comparable profiles support this assumption (Supplementary Table S13), missing-not-at-random mechanisms cannot be excluded.

## Conclusion

An inflammatory score derived from routine laboratory markers was independently associated with hematoma expansion and 90-day outcomes after intracerebral hemorrhage. When added to established HE and outcome models, it produced modest, reproducible improvements in discrimination, calibration, and reclassification. The score may serve as an adjunct for risk stratification; prospective external validation and decision-analytic studies are needed to establish clinical utility.

## Data availability

The datasets generated and/or analyzed during the current study are available from the corresponding author on reasonable request.

## Ethics approval and consent to participate

This study was approved by the Institutional Ethics Committee of Huazhong University of Science and Technology. All procedures were conducted in accordance with the Declaration of Helsinki and relevant institutional guidelines. Written informed consent was obtained from all participants prior to enrollment. The study was registered with the Chinese Clinical Trial Registry on 24 October 2020 (http://www.chictr.org.cn; registration number: ChiCTR-ROC2000039365).

## Consent for publication

Not applicable.

## Authors' contributions

Xianxian Li: Handled conceptualization; Methodology; Data curation; Analysis; Investigation, and manuscript writing. Yuchao Jia: Conducted analysis; Manuscript revision, and provided funding support. Jiahe Ye, Xin Liu, Yuanbing Lu, Haoyi Chen, Dan Zhang, Ruizhi Xiao, and Xiaodong Ye performed data collection (with Xiaodong Ye also providing funding); Shanshan Huang oversaw conceptualization, methodology, project supervision, manuscript revision, and funding; and Suiqiang Zhu provided supervision and project administration.

## Funding

This work was supported by the Noncommunicable Chronic Diseases-10.13039/501100018537National Science and Technology Major Project (Grant n°2023ZD0515600), the 10.13039/501100001809National Natural Science Foundation of China (Grant n°82401567), the 10.13039/501100003819Natural Science Foundation of Hubei Province (Grant n°2025AFB499), and the Science Foundation of Tongji Hospital (Grant n°2024A24).

## Declaration of competing interest

The authors declare no conflicts of interest.

## Appendix Supplementary materials

Image, application 1

## Footnotes

Supplementary material associated with this article can be found, in the online version, at doi:10.1016/j.clinsp.2026.101004.
