# Supplementary material for: A novel inflammatory score predicts hematoma expansion and 90‑day functional outcomes after spontaneous intracerebral hemorrhage
Source: Clinics (Sao Paulo). 2026 Jun 1;81:101004. doi: 10.1016/j.clinsp.2026.101004 (PMC13241867; doi:10.1016/j.clinsp.2026.101004)

**CLINICS-D-25-00983**

**Supplemental Files**

Supplementary Table S1 Center-specific reference intervals and derived cutoffs for the inflammatory score.

Supplementary Table S2 Comparison of the baseline characteristics between included and excluded patients.

Supplementary Table S3 Missing data and imputation bounds for baseline variables in the 3-month modified Rankin Scale and hematoma expansion cohorts.

Supplementary Table S4 Adjusted odds ratios for hematoma expansion in complete case analysis with and without the inflammatory score.

Supplementary Table S5 Complete-case sensitivity analysis of model performance for hematoma expansion and 3-month modified Rankin Scale outcomes.

Supplementary Table S6 Baseline characteristics and SMD comparing patients with and without follow-up CT (unweighted and after stabilized IPW).

Supplementary Table S7 Model performance for hematoma expansion under landmark (≤6 hours) and stabilized inverse probability weighting analyses.

Supplementary Table S8 Operating characteristics at prespecified risk thresholds for hematoma expansion and 3-month modified Rankin Scale outcomes.

Supplementary Table S9 Multicollinearity diagnostics (variance inflation factors) for score-augmented models.

Supplementary Table S10 Sensitivity analyses excluding baseline hematoma volume.

Supplementary Table S11 Adjusted odds ratios for 3-month modified Rankin Scale outcomes in the complete case sensitivity analysis.

Supplementary Table S12 Model performance for 3-month modified Rankin Scale outcomes (landmark analysis with laboratory tests obtained within 6 hours of symptom onset).

Supplementary Table S13 Baseline characteristics by missingness of inflammatory biomarkers.

Supplementary Figure S1 Availability of follow-up CT (24-48 h) and 90-day follow-up.

Supplementary Figure S2 ROC curves predicting hematoma expansion (n = 1005).

Supplementary Figure S3 Decision curve analysis for hematoma expansion and 90-day outcomes.

Supplementary Figure S4 ROC curves for 3-month functional outcome (n = 919).

Supplementary Figure S5 ROC curves predicting 3-month mortality (n = 919).

**Supplementary Table S1** Center-specific reference intervals and derived cutoffs for the inflammatory score.

| **Indicator** | **Reference interval (our center)** | **Derivation formula (using lab limits)** | **Cut-off used in this study** | **Points** |
| --- | --- | --- | --- | --- |
| **Neutrophils, % (n %)** | 40%‒75% | NLR ≥1.4×N%(UNL)/L%(LNL) | NLR ≥5.25 | Two points are assigned to the first positive indicator; each extra positive adds one point (0‒5 total) |
| **Lymphocytes, % (L%)** | 20%‒50% | ‒ | ‒ |  |
| **Monocytes, % (M%)** | 3%‒10% | MLR≥M%(UNL)/L%(LNL) | MLR ≥0.5 |  |
| **Platelets (PLT), ×10⁹/L** | 125‒350 | PLR≥PLT(UNL)/[WBC(UNL)*L%(LNL)] | PLR ≥184 |  |
| **SII** | ‒ | SII≥0.8×PLT(UNL)×N%(UNL)/L%(LNL) | SII ≥1050×10^9^/L |  |
| **LDH, U/L** | 120‒250 | LDH ≥0.9(UNL) | LDH ≥225 U/L | 2 |
| **hsCRP, mg/L** | 0‒10 | hsCRP ≥UNL | hsCRP ≥10 mg/L | 2 |

Ratios: NLR = N/L, MLR = M/L, SII = PLT×(N/L), PLR = PLT / (WBC×L%).

WBC reference interval at our center: (3.5‒9.5)×10⁹/L.

Cutoffs were derived from our center’s UNL/LNL using the stated formulas; other centers can recalculate them by substituting their own limits.

hsCRP, high-sensitivity C-Reactive Protein; LDH, Lactate Dehydrogenase; LNL, Lower Normal Limit; MLR, Monocyte-to-Lymphocyte Ratio; NLR, Neutrophil-to-Lymphocyte Ratio; PLR, Platelet-to-Lymphocyte Ratio; PLT, Platelets; SII, Systemic Immune-Inflammation Index; UNL, Upper Normal Limit; U/L, Units Per Liter; WBC, White Blood Cell Count.

**Supplementary Table S2** Comparison of the baseline characteristics between included and excluded patients.

|  | **Excluded (n = 1041)** | **Included (n = 1047)** | **p-value** |
| --- | --- | --- | --- |
| **Male sex, n (%)** | 685 (65.8%) | 710 (67.8%) | 0.329 |
| **Age, mean ± SD, y** | 55.51±11.88 | 56.21±12.51 | 0.191 |
| **Hypertension, n (%)** | 775 (74.4%) | 761 (72.7%) | 0.361 |
| **Diabetes mellitus, n (%)** | 106 (10.2%) | 113 (10.8%) | 0.649 |
| **Ischemic heart disease, n (%)** | 46 (4.4%) | 66 (6.3%) | 0.056 |
| **Hyperlipidemia, n (%)** | 51 (4.9%) | 65 (6.2%) | 0.192 |
| **Antiplatelets/Anticoagulants use, n (%)** | 74 (7.1%) | 84 (8%) | 0.430 |
| **Prior ischemic/ICH stroke, n (%)** | 130 (12.5%) | 153 (14.6%) | 0.156 |
| **Smoking, n (%)** | 337 (32.4%) | 357 (34.1%) | 0.403 |
| **Alcohol use, n (%)** | 297 (28.5%) | 328 (31.3%) | 0.163 |
| **Systolic blood pressure, mean±SD, mmHg** | 155.58±24.74 | 155.62±23.62 | 0.977 |
| **Diastolic blood pressure, mean±SD, mmHg** | 92.21±16.17 | 91.27±16.12 | 0.180 |
| **NIHSS on admission, median (IQR), score** | 11 (4-19) | 11 (6-17) | 0.341 |
| **ICH location, n (%)** |  |  | 0.470 |
| Lobar | 285 (27.4%) | 272 (26%) |  |
| Subcortical | 756 (72.6%) | 775 (74%) |  |
| **Presence of IVH, n (%)** | 354 (34%) | 332 (31.7%) | 0.264 |
| **Treatment, n (%)** |  |  | 0.689 |
| Medical treatment | 745 (71.6%) | 741 (70.8%) |  |
| Surgical management | 296 (28.4%) | 306 (29.2%) |  |

Baseline characteristics are summarized from observed data only; imputed values were not used. Values are mean±SD or median (IQR) as appropriate; categorical variables are n (%).

ICH, Intracerebral Hemorrhage; IQR, Interquartile Range; IVH, Intraventricular Hemorrhage; NIHSS, National Institute of Health Stroke Scale; p-value, Probability value; SD, Standard Deviation.

**Supplementary Table S3** Missing data and imputation bounds for baseline variables in the 3-month modified Rankin Scale and hematoma expansion cohorts.

| **Variable** | **3-month mRS (n = 953)** | | **HE (n = 1047)** | |
| --- | --- | --- | --- | --- |
|  | **Bounds^c^** | **Missing before MI, n (%)** | **Bounds^c^** | **Missing before MI, n (%)** |
| **Core laboratory variables (MI)** |  |  |  |  |
| **White blood cell count^a^** | 0.88‒27.69 | 5 (0.52%) | 0.94‒27.22 | 6 (0.57%) |
| **Neutrophil count^a^** | 0.00‒25.59 | 5 (0.52%) | 0.06‒25.19 | 6 (0.57%) |
| **Lymphocyte count^a^** | 0.00‒4.47 | 6 (0.63%) | 0.00‒4.47 | 7 (0.67%) |
| **Monocyte count^a^** | 0.00‒1.98 | 5 (0.52%) | 0.00‒1.89 | 6 (0.57%) |
| **Platelet count^a^** | 1.10‒458.16 | 3 (0.31%) | 1.10‒457.11 | 4 (0.38%) |
| **hsCRP^a^** | 0.00‒282.69 | 16 (1.68%) | 0.00‒274.21 | 20 (1.91%) |
| **LDH^a^** | 78.68‒594.99 | 11 (1.15%) | 80.00‒583.98 | 15 (1.43%) |
| **Non-core variables (not imputed)** |  |  |  |  |
| **Age, y^b^** | ‒ | 0 | ‒ | 0 |
| **Systolic blood pressure, mmHg^b^** | ‒ | 0 | ‒ | 0 |
| **Diastolic blood pressure, mmHg^b^** | ‒ | 0 | ‒ | 0 |
| **Hb, g/L^b^** | ‒ | 4 (0.42%) | ‒ | 6 (0.58%) |
| **ALT, U/L^b^** | ‒ | 1 (0.11%) | ‒ | 2 (0.19%) |
| **AST, U/L^b^** | ‒ | 1 (0.11%) | ‒ | 2 (0.19%) |
| **Creatinine, μmol/L^b^** | ‒ | 13 (1.38%) | ‒ | 17 (1.65%) |
| **Uric acid, mmol/L^b^** | ‒ | 2 (0.21%) | ‒ | 4 (0.38%) |
| **International normalized ratio^b^** | ‒ | 20 (2.14%) | ‒ | 25 (2.45%) |
| **Glucose level, mmol/L^b^** | ‒ | 117 (14%) | ‒ | 125 (13.56%) |
| **NIHSS on admission, score^b^** | ‒ | 0 | ‒ | 0 |
| **Baseline ICH volume, mL^b^** | ‒ | 0 | ‒ | 0 |
| **Time from symptom onset to CT, h^b^** | ‒ | ‒ | ‒ | 0 |

^a^ Seven laboratory variables (white blood cell, neutrophil, lymphocyte, monocyte, platelet counts; hsCRP; LDH) were imputed using multiple imputation by chained equations (MICE; m = 20). Predictive mean matching (PMM; k = 15) was the default; Multiple Imputation using Distance Aided Selection (MIDAS)-touch (≤ 25 donors) or normal regression was used only if PMM failed to yield an in-bounds draw. Plausibility bounds ‒ observed 0.5^th^/99.5^th^ percentiles expanded by 10% (with a lower bound of 0 for non-negative biomarkers) ‒ were applied to imputed values only. A bounded donor hot-deck replaced any residual missing or out-of-bounds draws. After imputation, all analysis rows were complete for these seven laboratory variables.

^b^ Not imputed; observed data only. Bounds are not applicable.

^c^ Bounds are cohort‑specific (mRS cohort | HE cohort). Lower truncation at 0 applies only to imputed variables.

ALT, Alanine Aminotransferase; AST, Aspartate Aminotransferase; CT, Computed Tomography; Hb, Hemoglobin; hsCRP, high-sensitivity C-Reactive Protein; ICH, Intracerebral Hemorrhage; LDH, Lactate Dehydrogenase; MI, Multiple Imputation; mRS, modified Rankin Scale; NIHSS, National Institutes of Health Stroke Scale.

**Supplementary Table S4** Adjusted odds ratios for hematoma expansion in complete case analysis with and without the inflammatory score.

| **Variable** | **HE (n = 1005)** | |
| --- | --- | --- |
|  | **Adjusted Odds ratio (95% CI)** | **p-value** |
| **5-predictor model** |  |  |
| Antiplatelets use, n (%) | 2.46 (1.25‒4.84) | 0.009 |
| Anticoagulants use, n (%) | 2.44 (1.30‒4.60) | 0.006 |
| Time from symptom onset to CT, h | 0.84 (0.78‒0.91) | <0.001 |
| Baseline ICH volume, mL | 1.01 (1.00‒1.01) | 0.001 |
| NCCT hypodensities, n (%) | 3.17 (2.22‒4.53) | <0.001 |
| **5-predictor model plus inflammatory score** |  |  |
| Antiplatelets use, n (%) | 2.84 (1.42‒5.71) | 0.003 |
| Anticoagulants use, n (%) | 2.77 (1.45‒5.31) | 0.002 |
| Time from symptom onset to CT, h | 0.84 (0.78‒0.91) | <0.001 |
| Baseline ICH volume, mL | 1.00 (1.00‒1.01) | 0.196 |
| NCCT hypodensities, n (%) | 3.13 (2.17‒4.50) | <0.001 |
| Inflammatory score, score | 1.19 (1.12‒1.27) | <0.001 |

Complete-case analysis excluded participants missing inflammatory score components.

CI, Confidence Interval; CT, Computed Tomography; ICH, Intracerebral Hemorrhage; IVH, Intraventricular Hemorrhage; NCCT, Non-Contrast Computed Tomography; p-value, Probability value.

**Supplementary Table S5** Complete-case sensitivity analysis of model performance for hematoma expansion and 3-month modified Rankin Scale outcomes.

| **Model** | **AUC (95% CI)** | **ΔAUC** | **DeLong *P*** | **Wald *P*** | **LRT *P*** | **Brier** | **cfNRI (95% CI; p)** | **IDI (95% CI; p)** |
| --- | --- | --- | --- | --- | --- | --- | --- | --- |
| **HE (n = 1005)** |  |  |  |  |  |  |  |  |
| 5-predictor model | 0.680 (0.644‒0.716) |  |  |  |  | 0.189 |  |  |
| 5-predictor model plus Inflammatory Score | 0.709 (0.674‒0.745) | 0.029 | 0.007 | NA | <0.001 | 0.182 | 0.394 (0.301‒0.487; <0.001) | 0.031 (0.020‒0.042; <0.001) |
| **3-month mRS 4‒6 (n = 919)** | | | | | | | | |
| max-ICH components model | 0.831 (0.805‒0.857) |  |  |  |  | 0.167 |  |  |
| max-ICH components model plus inflammatory score | 0.847 (0.822‒0.872) | 0.016 | 0.020 | NA | <0.001 | 0.157 | 0.465 (0.375‒0.555; <0.001) | 0.040 (0.027‒0.053; <0.001) |
| **3-month mRS 6 (n = 919)** |  |  |  |  |  |  |  |  |
| max-ICH components model | 0.823 (0.781‒0.865) |  |  |  |  | 0.087 |  |  |
| max-ICH components model plus inflammatory score | 0.848 (0.813‒0.883) | 0.025 | 0.024 | NA | <0.001 | 0.083 | 0.557 (0.423‒0.691; <0.001) | 0.036 (0.019‒0.052; <0.001) |

Complete-case analysis excluded participants missing inflammatory score components.

AUC, Area Under the receiver operating Characteristic Curve; Brier, Brier score (mean squared error of probabilistic predictions; lower values indicate better accuracy); CI, Confidence Interval; cfNRI, category-free Net Reclassification Improvement (i.e., continuous NRI that does not require prespecified risk categories); DeLong *P*, two-sided p-value from DeLong’s test comparing paired AUCs; ΔAUC, difference in AUC between the augmented model (with the inflammatory score) and the base model; IDI, Integrated Discrimination Improvement; LRT *P*, p-value from the likelihood-ratio test (not applicable for multiply imputed models); mRS, modified Rankin Scale; NA, Not Applicable.

**Supplementary Table S6** Baseline characteristics and SMD comparing patients with and without follow-up CT (unweighted and after stabilized IPW).

| **Variable** | **Unweighted** | | | **SMD after CT-IPW** |
| --- | --- | --- | --- | --- |
|  | **Follow-up CT obtained (n = 1047)** | **No follow-up CT (n = 610)** | **SMD (Unweighted)** |  |
| **Age, mean±SD, y** | 56.21±12.51 | 55.56±11.67 | 0.054 | 0.004 |
| **Male sex, n (%)** | 710 (67.8%) | 408 (66.9%) | 0.02 | -0.002 |
| **Hypertension, n (%)** | 761 (72.7%) | 431 (70.7%) | 0.045 | -0.003 |
| **Diabetes mellitus, n (%)** | 113 (10.8%) | 51 (8.4%) | 0.083 | 0.002 |
| **Ischemic heart disease, n (%)** | 66 (6.3%) | 34 (5.6%) | 0.031 | 0.007 |
| **Hyperlipidemia, n (%)** | 65 (6.2%) | 26 (4.3%) | 0.087 | 0.009 |
| **Antiplatelets use, n (%)** | 45 (4.3%) | 31 (5.1%) | -0.037 | 0.006 |
| **Anticoagulants use, n (%)** | 39 (3.7%) | 20 (3.3%) | 0.024 | 0.012 |
| **Prior ischemic/ICH stroke, n (%)** | 153 (14.6%) | 76 (12.5%) | 0.063 | 0.001 |
| **Smoking, n (%)** | 357 (34.1%) | 201 (33%) | 0.024 | -0.004 |
| **Alcohol use, n (%)** | 328 (31.3%) | 177 (29%) | 0.05 | -0.002 |
| **Systolic blood pressure, mean±SD, mmHg** | 155.62±23.62 | 156.36±24.03 | -0.031 | -0.005 |
| **Diastolic blood pressure, mean±SD, mmHg** | 91.27±16.12 | 92.76±16.02 | -0.093 | -0.006 |
| **NIHSS on admission, median (IQR), score** | 11 (6‒17) | 11 (4‒18) | -0.004 | -0.009 |
| **Baseline ICH volume, median (IQR), mL** | 16.47 (7.31‒33.93) | 19.26 (9.93‒34.88) | -0.078 | 0.001 |
| **ICH location (lobar), n (%)** | 272 (26%) | 157 (25.7%) | 0.006 | -0.001 |
| **Presence of IVH, n (%)** | 332 (31.7%) | 189 (31%) | 0.016 | -0.003 |

Values are mean ± SD or median (IQR) as appropriate; categorical variables are n (%).

CT, Computed Tomography; IPW, Inverse Probability Weighting; IQR, Interquartile Range; IVH, Intraventricular Hemorrhage; NIHSS, National Institutes of Health Stroke Scale; SD, Standard Deviation; SMD, Standardized Mean Difference.

**Supplementary Table S7** Model performance for hematoma expansion under landmark (≤6-hours) and stabilized inverse probability weighting analyses.

| **Analysis** | **Model** | **N (events/non-events)** | **AUC (95% CI)** | **Brier** | **ΔAUC** | **DeLong *P*** | **Wald *P*** |
| --- | --- | --- | --- | --- | --- | --- | --- |
| Unweighted | 5-predictor model | 818 (228/590) | 0.676 (0.636‒0.717) | 0.186 |  |  |  |
|  | 5-predictor model plus inflammatory score | 818 (228/590) | 0.701 (0.660‒0.742) | 0.179 | 0.024 | 0.059 | <0.001 |
| E-IPW | 5-predictor model | 818 (228/590) | 0.677 (0.637‒0.717) | 0.186 |  |  |  |
|  | 5-predictor model plus inflammatory score | 818 (228/590) | 0.701 (0.659‒0.742) | 0.179 | 0.024 | 0.070 | <0.001 |
| CT-IPW | 5-predictor model | 818 (228/590) | 0.676 (0.636‒0.717) | 0.186 |  |  |  |
|  | 5-predictor model plus inflammatory score | 818 (228/590) | 0.701 (0.660‒0.742) | 0.179 | 0.025 | 0.053 | <0.001 |
| Combined IPW | 5-predictor model | 818 (228/590) | 0.677 (0.637‒0.717) | 0.186 |  |  |  |
|  | 5-predictor model plus inflammatory score | 818 (228/590) | 0.701 (0.660‒0.742) | 0.179 | 0.024 | 0.061 | <0.001 |

The 5-predictor model includes anticoagulant use, antiplatelet use, time from symptom onset to baseline computed tomography, baseline hematoma volume, and hypodensities on baseline computed tomography; the augmented model additionally includes the inflammatory score. AUCs and ΔAUC are computed from the mean predicted probabilities across imputations. AUC/Brier were evaluated on unweighted predictions; stabilized IPW (99th-percentile truncated) was applied at model fitting only.

AUC, Area Under the Receiver Operating Characteristic (ROC) Curve; Brier, Brier score (mean squared error of predicted probabilities; lower is better); CI, Confidence Interval; Combined IPW, product of E-IPW and CT-IPW; CT-IPW, stabilized Inverse Probability Weighting for receipt of a follow-up computed-tomography scan (reweights the landmark sample to the full inception cohort); DeLong *P*, two-sided p-value from DeLong's test comparing paired AUCs; E-IPW, Stabilized Inverse Probability Weighting for having laboratory tests drawn ≤6-hours after symptom onset (“early labs”); IPW, Inverse Probability Weighting; Landmark-analysis restricted to patients with laboratory tests obtained ≤6-hours after symptom onset.

**Supplementary Table S8** Operating characteristics at prespecified risk thresholds for hematoma expansion and 3-month modified Rankin Scale outcomes.

| **Outcome** | **Model group** | **Cutoff** | **Sensitivity, % (95% CI)** | **Specificity, % (95% CI)** | **PPV, % (95% CI)** | **NPV, % (95% CI)** | **Youden index** |
| --- | --- | --- | --- | --- | --- | --- | --- |
| **Main analysis (multiple imputation)** | | | | | | | |
| 3-month mRS 4‒6 (n = 953) | max-ICH components model | ≥0.30 | 84.5 (80.6‒87.7) | 64.4 (60.1‒68.3) | 63.1 (58.9‒67.1) | 85.2 (81.5‒88.3) | 0.489 |
|  | max-ICH components model plus inflammatory score | ≥0.30 | 85.7 (81.9‒88.8) | 65.0 (60.9‒68.8) | 63.8 (59.7‒67.8) | 86.3 (82.7‒89.3) | 0.507 |
| 3-month mRS 6 (n = 953) | max-ICH components model | ≥0.10 | 81.7 (73.8‒87.6) | 69.5 (66.3‒72.5) | 27.8 (23.4‒32.7) | 96.3 (94.5‒97.6) | 0.512 |
|  | max-ICH components model plus inflammatory score | ≥0.10 | 82.5 (74.7‒88.3) | 70.0 (66.8‒73.0) | 28.4 (23.9‒33.3) | 96.5 (94.7‒97.7) | 0.525 |
| HE (n = 1047) | 5-predictor model | ≥0.30 | 49.0 (43.4‒54.6) | 76.5 (73.3‒79.4) | 45.3 (40.0‒50.8) | 79.0 (75.9‒82.1) | 0.255 |
|  | 5-predictor model plus inflammatory score | ≥0.30 | 60.4 (54.8‒65.8) | 72.6 (69.3‒75.7) | 46.8 (41.8‒51.7) | 82.2 (79.1‒84.9) | 0.330 |
| **Sensitivity analysis (Complete-case)** | | | | | | | |
| 3-month mRS 4‒6 (n = 919) | max-ICH components model | ≥0.30 | 84.0 (80.0‒87.4) | 65.4 (61.2‒69.3) | 63.3 (59.0‒67.4) | 85.2 (81.4‒88.3) | 0.494 |
|  | max-ICH components model plus inflammatory score | ≥0.30 | 85.3 (81.4‒88.5) | 66.7 (63.3‒70.0) | 64.6 (60.0‒68.9) | 86.5 (82.0‒90.3) | 0.520 |
| 3-month mRS 6 (n = 919) | max-ICH components model | ≥0.10 | 78.4 (70.7‒84.6) | 71.8 (66.8‒74.8) | 27.6 (23.0‒32.8) | 96.0 (94.2‒97.3) | 0.502 |
|  | max-ICH components model plus inflammatory score | ≥0.10 | 82.0 (75.1‒87.2) | 72.6 (69.5‒75.7) | 32.8 (28.0‒37.9) | 96.7 (95.0‒97.9) | 0.546 |
| HE (n = 1005) | 5-predictor model | ≥0.30 | 51.2 (45.5‒56.8) | 75.5 (72.2‒78.5) | 46.5 (41.1‒51.9) | 78.8 (75.6‒81.7) | 0.267 |
|  | 5-predictor model plus inflammatory score | ≥0.30 | 62.4 (56.7‒67.7) | 70.4 (67.0‒73.7) | 46.7 (41.8‒51.6) | 81.8 (78.6‒84.7) | 0.328 |

HE models: the base model includes anticoagulant use, antiplatelet use, time from symptom onset to computed tomography, baseline hematoma volume, and hypodensities; the augmented model adds the inflammatory score. 3-month mRS models: the base model includes age, baseline hematoma volume, lobar location, intraventricular hemorrhage, National Institutes of Health Stroke Scale at admission, and anticoagulant use; the augmented model adds the inflammatory score. Risk thresholds were prespecified at 0.10, 0.20, and 0.30, and only the threshold that maximized the Youden index is shown.

CI, Confidence Interval; HE, Hematoma Expansion; mRS, modified Rankin Scale; NPV, Negative Predictive Value; PPV, Positive Predictive Value.

**Supplementary Table S9** Multicollinearity diagnostics (variance inflation factors) for score-augmented models.

| **Outcome** | **VIF (baseline hematoma volume)** | **VIF (inflammatory score)** | **Maximum VIF** |
| --- | --- | --- | --- |
| **Main analysis (multiple imputation)** | | | |
| 3-month mRS 4‒6 (n = 953) | 1.508 (1.506–1.509) | 1.231 (1.228–1.237) | 1.509 |
| 3-month mRS 6 (n = 953) | 1.508 (1.506–1.509) | 1.231 (1.228–1.237) | 1.509 |
| HE (n = 1047) | 1.159 (1.156–1.161) | 1.126 (1.123–1.128) | 1.161 |
| **Sensitivity analysis (complete-case)** | | | |
| 3-month mRS 4‒6 (n = 919) | 1.503 | 1.235 | 1.503 |
| 3-month mRS 6 (n = 919) | 1.503 | 1.235 | 1.503 |
| HE (n = 1005) | 1.165 | 1.129 | 1.165 |

VIFs were calculated for the prespecified score-augmented models. In multiple imputation analyses, VIFs are summarized across imputations as mean (min–max); complete-case analyses report single-fit VIFs.

HE, Hematoma Expansion; mRS, modified Rankin Scale; VIF, Variance Inflation Factor.

**Supplementary Table S10** Sensitivity analyses excluding baseline hematoma volume.

| **Outcome** | **N (events/non-events)** | **AUC base (no volume)** | **AUC + score (no volume)** | **ΔAUC** | **DeLong *P*** | **Inflammatory score OR (95% CI)** | **Score *P*** |
| --- | --- | --- | --- | --- | --- | --- | --- |
| **Main analysis (multiple imputation)** | | | | | | | |
| 3-month mRS 4‒6 (n = 953) | 953 (399/554) | 0.821 | 0.840 | 0.019 | 0.007 | 1.29 (1.20–1.38) | <0.001 |
| 3-month mRS 6 (n = 953) | 953 (120/833) | 0.808 | 0.833 | 0.025 | 0.026 | 1.32 (1.19–1.46) | <0.001 |
| HE (n = 1047) | 1047 (298/749) | 0.666 | 0.706 | 0.040 | 0.002 | 1.20 (1.13–1.27) | <0.001 |
| **Sensitivity analysis (complete-case)** | | | | | | | |
| 3-month mRS 4‒6 (n = 919) | 919(382/537) | 0.825 | 0.844 | 0.020 | 0.009 | 1.30 (1.21–1.40) | <0.001 |
| 3-month mRS 6 (n = 919) | 919(111/808) | 0.812 | 0.842 | 0.031 | 0.010 | 1.36 (1.22–1.52) | <0.001 |
| HE (n = 1005) | 1005(295/710) | 0.666 | 0.707 | 0.041 | 0.001 | 1.20 (1.13–1.28) | <0.001 |

Base models are identical to the prespecified models except that baseline hematoma volume is removed. ORs are for the inflammatory score per 1-point increase in the score-augmented model.

AUC, Area Under the Receiver Operating Characteristic Curve; CI, Confidence Interval; HE, Hematoma Expansion; mRS, modified Rankin Scale; OR, Odds Ratio.

**Supplementary Table S11** Adjusted odds ratios for 3-month modified Rankin Scale outcomes in the complete case sensitivity analysis

| **Variable** | **3-month mRS 4‒6 (n = 919)** | | **3-month mRS 6 (n = 919)** | |
| --- | --- | --- | --- | --- |
|  | **Adjusted Odds ratio (95% CI)** | **p-value** | **Adjusted Odds ratio (95%CI)** | **p-value** |
| **max-ICH components model** |  |  |  |  |
| Age, year | 1.04 (1.03‒1.06) | <0.001 | 1.05 (1.03‒1.06) | <0.001 |
| Anticoagulants use, n (%) | 0.73 (0.32‒1.64) | 0.441 | 0.91 (0.32‒2.59) | 0.853 |
| NIHSS on admission, score | 1.13 (1.11‒1.16) | <0.001 | 1.10 (1.07‒1.12) | <0.001 |
| ICH location (lobar), n (%) | 1.56 (1.03‒2.37) | 0.037 | 1.18 (0.68‒2.05) | 0.564 |
| Baseline ICH volume, mL | 1.02 (1.01‒1.03) | <0.001 | 1.01 (1.00‒1.02) | 0.002 |
| Presence of IVH, n (%) | 1.80 (1.28‒2.55) | <0.001 | 1.12 (0.70‒1.80) | 0.627 |
| **max-ICH components model plus inflammatory score** |  |  |  |  |
| Age, year | 1.04 (1.03‒1.06) | <0.001 | 1.05 (1.03‒1.07) | <0.001 |
| Anticoagulants use, n (%) | 0.96 (0.42‒2.21) | 0.931 | 1.14 (0.38‒3.45) | 0.816 |
| NIHSS on admission, score | 1.12 (1.09‒1.14) | <0.001 | 1.08 (1.06‒1.11) | <0.001 |
| ICH location (lobar), n (%) | 1.73 (1.12‒2.67) | 0.013 | 1.36 (0.77‒2.40) | 0.291 |
| Baseline ICH volume, mL | 1.01 (1.00‒1.02) | 0.002 | 1.01 (1.00‒1.02) | 0.019 |
| Presence of IVH, n (%) | 1.71 (1.20‒2.44) | 0.003 | 1.06 (0.65‒1.71) | 0.820 |
| Inflammatory score, score | 1.28 (1.19‒1.38) | <0.001 | 1.34 (1.20‒1.50) | <0.001 |

Complete-case analysis excluded participants missing inflammatory score components.

CI, Confidence Interval; ICH, Intracerebral Hemorrhage; IQR, Interquartile Range; IVH, Intraventricular Hemorrhage; mRS, modified Rankin Scale; NIHSS, National Institutes of Health Stroke Scale; p-value, Probability value.

**Supplementary Table S12** Model performance for 3-month modified Rankin Scale outcomes (landmark analysis with laboratory tests obtained within 6-hours of symptom onset).

| **Analysis** | **Model** | **N (events/non-events)** | **AUC (95% CI)** | **Brier** | **ΔAUC** | **DeLong p** | **Wald p** |
| --- | --- | --- | --- | --- | --- | --- | --- |
| 3-month mRS 4‒6 (Unweighted) | max-ICH components model | 733 (311/422) | 0.816 (0.785‒0.847) | 0.173 |  |  |  |
|  | max-ICH components model plus inflammatory score | 733 (311/422) | 0.832 (0.803‒0.862) | 0.164 | 0.017 | 0.035 | <0.001 |
| 3-month mRS 6 (Unweighted) | max-ICH components model | 733 (96/637) | 0.812 (0.766‒0.858) | 0.096 |  |  |  |
|  | max-ICH components model plus inflammatory score | 733 (96/637) | 0.831 (0.792‒0.871) | 0.091 | 0.020 | 0.147 | <0.001 |

The max-ICH components model includes six prespecified predictors: age; baseline hematoma volume; lobar location; intraventricular hemorrhage; National Institutes of Health Stroke Scale score at admission; and preadmission anticoagulant use. The augmented model additionally includes the inflammatory score. AUC and ΔAUC are computed from the mean of the predicted probabilities across imputations.

AUC, Area Under the Receiver-Operating-Characteristic Curve; Brier, Brier score (mean squared error of probabilistic predictions; lower values indicate better accuracy); CI, Confidence Interval; ΔAUC, change in AUC between the model that adds the inflammatory score and the base model; DeLong p, two-sided p-value from DeLong’s test comparing paired AUCs; ICH, Intracerebral Hemorrhage; Landmark, analysis restricted to patients whose laboratory tests were obtained within 6-hours after symptom onset; mRS, modified Rankin Scale.

**Supplementary Table S13** Baseline characteristics by missingness of inflammatory biomarkers.

| **Variable** | **Total**  **(n = 1047)** | **No missing biomarker**  **(n = 1005)** | **Any missing biomarker**  **(n = 42)** | **SMD** |
| --- | --- | --- | --- | --- |
| **Male sex, n (%)** | 710 (67.8%) | 685 (68.2%) | 25 (59.5%) | 0.180 |
| **Age, mean±SD, y** | 56.21±12.51 | 56.14±12.45 | 57.76±13.98 | 0.122 |
| **Hypertension, n (%)** | 761 (72.7%) | 727 (72.3%) | 34 (81.0%) | 0.205 |
| **Diabetes mellitus, n (%)** | 113 (10.8%) | 108 (10.7%) | 5 (11.9%) | 0.037 |
| **Ischemic heart disease, n (%)** | 66 (6.3%) | 62 (6.2%) | 4 (9.5%) | 0.125 |
| **Antiplatelets use, n (%)** | 45 (4.3%) | 44 (4.4%) | 1 (2.4%) | 0.111 |
| **Anticoagulants use, n (%)** | 39 (3.7%) | 38 (3.8%) | 1 (2.4%) | 0.081 |
| **Prior ischemic/ICH stroke, n (%)** | 153 (14.6%) | 150 (14.9%) | 3 (7.1%) | 0.250 |
| **Smoking, n (%)** | 357 (34.1%) | 346 (34.4%) | 11 (26.2%) | 0.180 |
| **Alcohol use, n (%)** | 328 (31.3%) | 318 (31.6%) | 10 (23.8%) | 0.176 |
| **Time from symptom onset to CT, median (IQR), h** | 3.00 (2.00‒5.50) | 3.00 (2.00‒5.50) | 2.85 (1.62‒5.00) | 0.050 |
| **Baseline ICH volume, median (IQR), mL** | 16.47 (7.33‒33.92) | 16.69 (7.41‒33.92) | 13.61 (6.68‒34.63) | 0.133 |
| **Systolic blood pressure, mean±SD, mmHg** | 155.61±23.62 | 155.70±23.57 | 153.52±24.91 | 0.090 |
| **Diastolic blood pressure, mean±SD, mmHg** | 91.26±16.11 | 91.32±16.18 | 89.74±14.49 | 0.103 |
| **NIHSS on admission, median (IQR), score** | 11.00 (6.00‒17.00) | 11.00 (6.00‒18.00) | 9.00 (4.00‒15.00) | 0.269 |
| **ICH location (Subcortical), n (%)** | 775 (74.0%) | 744 (74.0%) | 31 (73.8%) | 0.005 |
| **Presence of IVH, n (%)** | 332 (31.7%) | 319 (31.7%) | 13 (31.0%) | 0.017 |
| **NCCT hypodensities, n (%)** | 171 (16.3%) | 164 (16.3%) | 7 (16.7%) | 0.009 |

Values are mean ± SD or median (IQR) as appropriate; categorical variables are n (%).

CT, Computed Tomography; ICH, Intracerebral Hemorrhage; IQR, Interquartile Range; IVH, Intraventricular Hemorrhage; NCCT, Non-Contrast Computed Tomography; NIHSS, National Institute of Health Stroke Scale; SD, Standard Deviation; SMD, Standardized Mean Difference.

**Supplementary Figure S1** Availability of follow-up CT (24‒48h) and 90-day follow-up. CT, Computed Tomography; mRS, modified Rankin Scale.


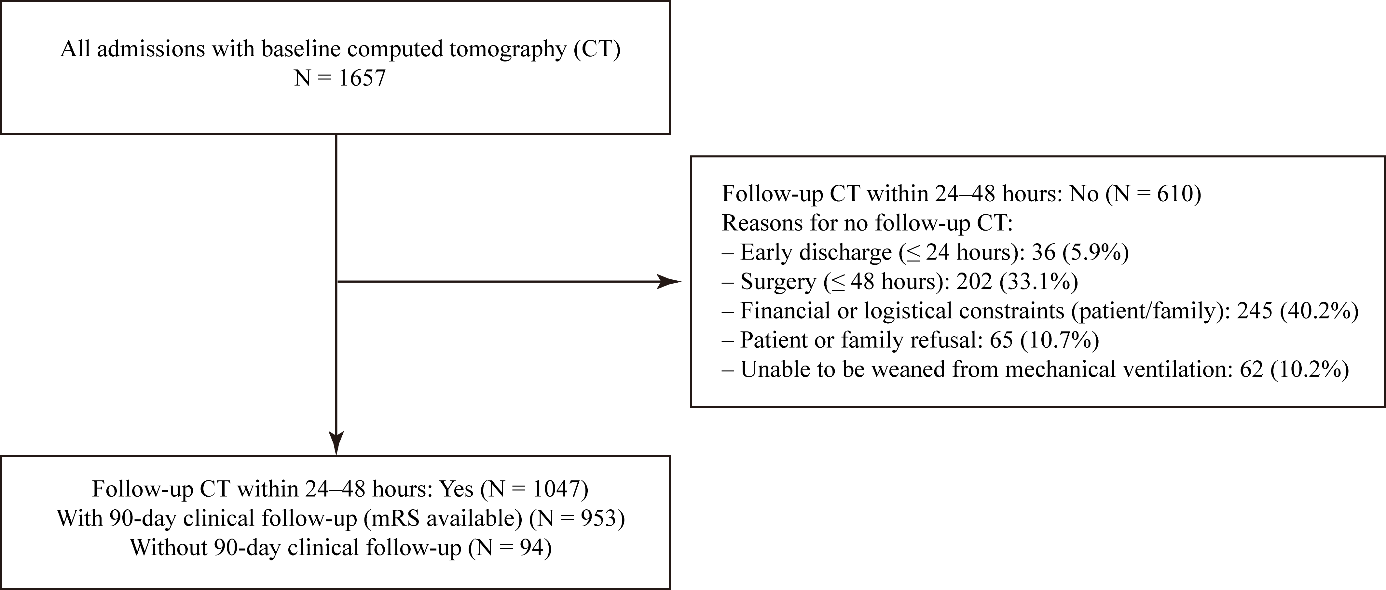


**Supplementary Figure S2** ROC curves predicting hematoma expansion (n = 1005). The 5-predictor model includes anticoagulant use, antiplatelet use, time from symptom onset to baseline computed tomography, baseline hematoma volume, and hypodensities on baseline computed tomography; the augmented model additionally includes the inflammatory score. Complete-case analysis excluded participants missing inflammatory score components. Shaded areas indicate 95% confidence bands for the ROC curves. AUC, Area Under the Curve; *P*, Probability value; ROC, Receiver Operating Characteristic.


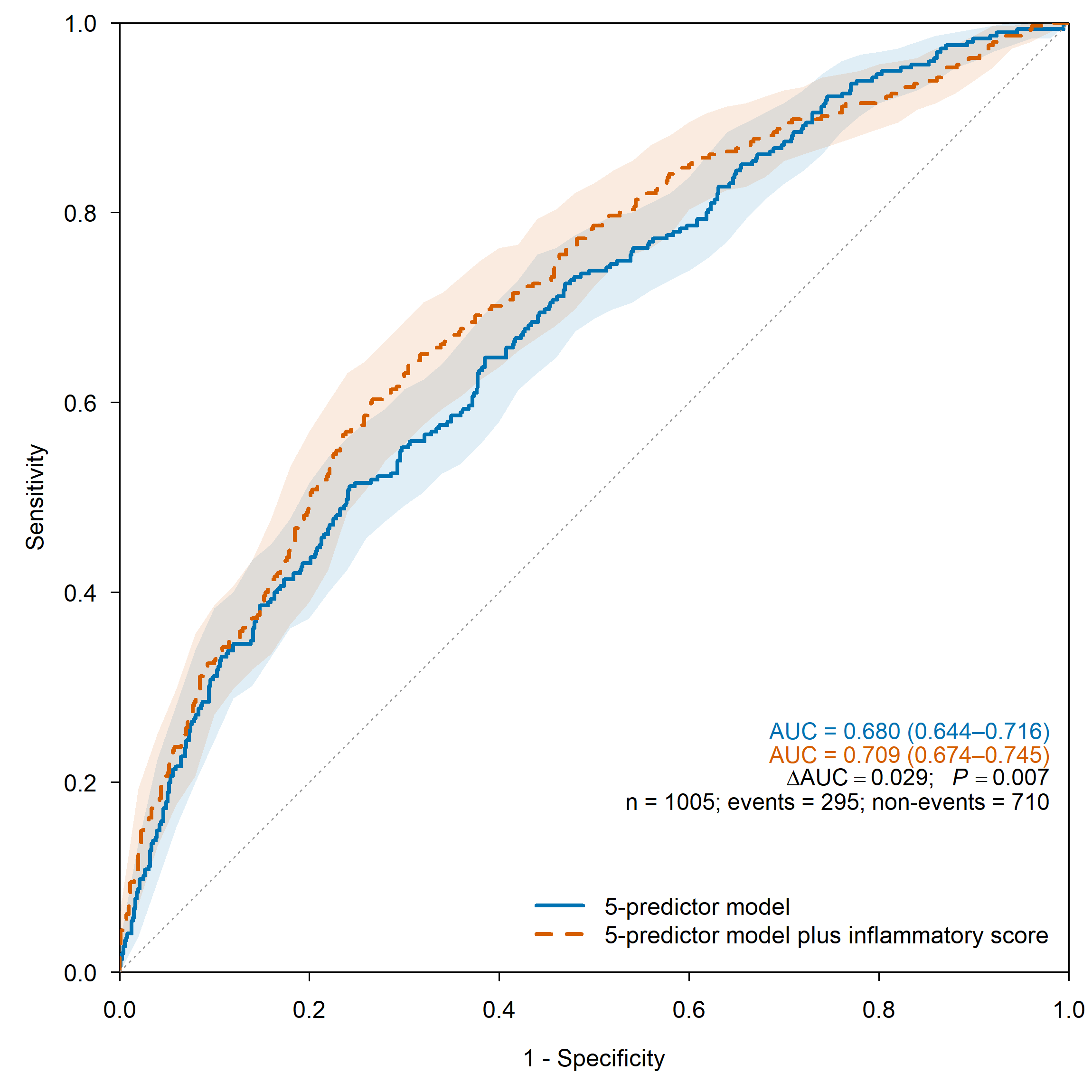


**Supplementary Figure S3** Decision curve analysis for hematoma expansion and 90-day outcomes. Decision curve analysis comparing the base models with and without the composite inflammatory score across threshold probabilities from 0.05 to 0.35. For hematoma expansion, the base model included five predictors; for 90-day outcomes, the base model included components of the maximum intracerebral hemorrhage score. Panels A–C show the primary analyses using multiple imputation for hematoma expansion, 90-day modified Rankin Scale score 4–6, and 90-day mortality. Panels D–F show the corresponding complete-case analyses. Net benefit is shown for each model, alongside treat-all and treat-none strategies. HE, Hematoma Expansion; mRS, modified Rankin Scale.


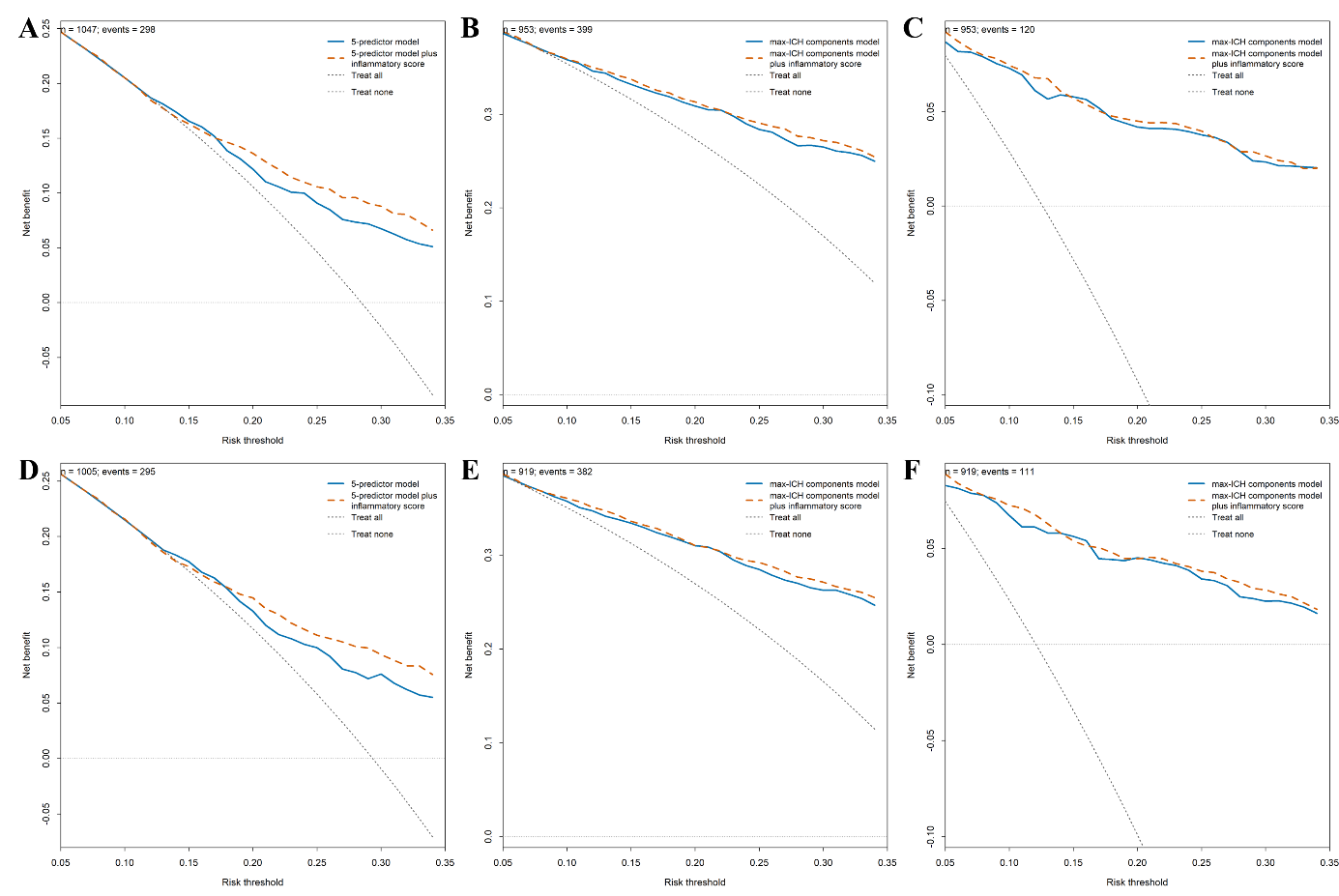


**Supplementary Figure S4** ROC curves for 3-month functional outcome (n = 919). The max-ICH components model includes six prespecified predictors: age; baseline hematoma volume; lobar location; intraventricular hemorrhage; National Institutes of Health Stroke Scale score at admission; and preadmission anticoagulant use. The augmented model additionally includes the inflammatory score. Complete-case analysis excluded participants missing inflammatory score components. Shaded areas indicate 95% confidence bands for the ROC curves. AUC, Area Under the Curve; *P*, Probability value; ROC, Receiver Operating Characteristic.


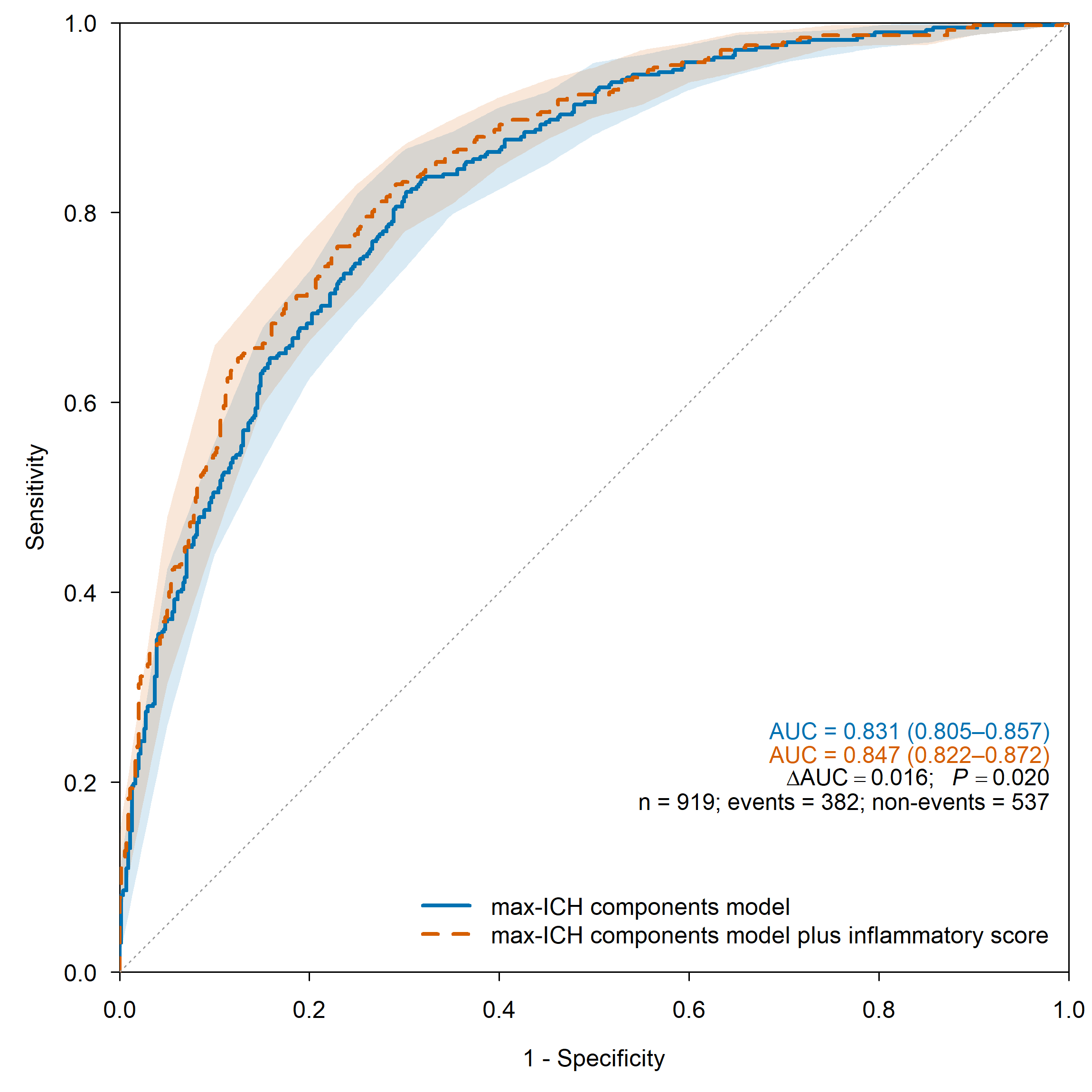


**Supplementary Figure S5** ROC curves predicting 3-month mortality (n = 919). The max-ICH components model includes six prespecified predictors: age; baseline hematoma volume; lobar location; intraventricular hemorrhage; National Institutes of Health Stroke Scale score at admission; and preadmission anticoagulant use. The augmented model additionally includes the inflammatory score. Complete-case analysis excluded participants missing inflammatory score components. Shaded areas indicate 95% confidence bands for the ROC curves. AUC, Area Under the Curve; *P*, Probability value; ROC, Receiver Operating Characteristic.


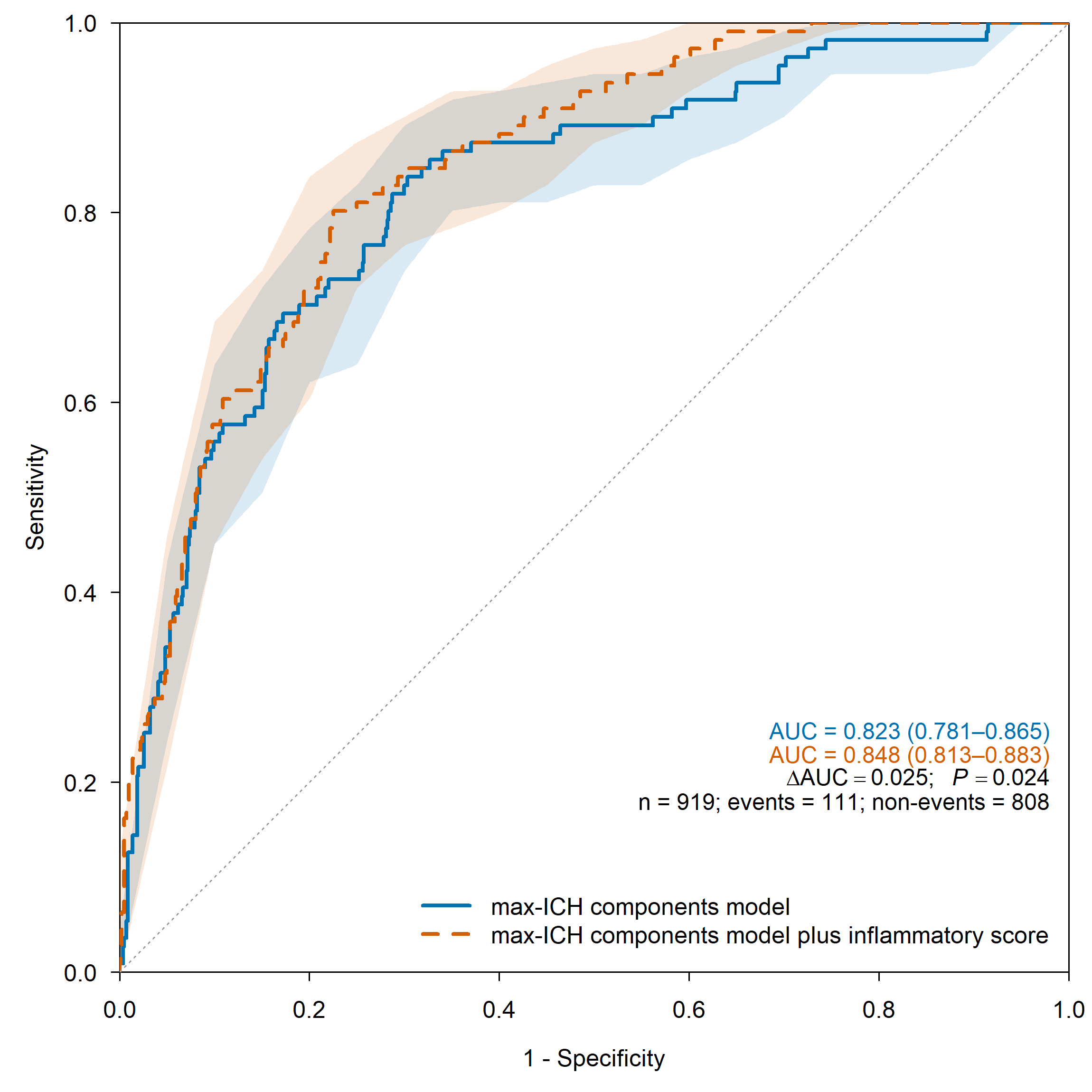

Supplement: Supplementary file 1 [file mmc1.docx]
